# Note on Particle Velocity in Collisions Between Liquid Drops and Solids

**DOI:** 10.6028/jres.064A.048

**Published:** 1960-12-01

**Authors:** Olive G. Engel

## Abstract

Equations are developed for plane-wave particle velocity produced in solid-against-liquid collisions. An explicit expression for the dimensionless coefficient *α* that appears in these equations is deduced.

Collisions between liquid drops and the planar surfaces of solids have become important in the present era of high-speed flight. Except for the pressure that results when a drop of incompressible liquid collides with the planar surface of an unyielding solid [1],^1^ exact hydrodynamic treatments of the various aspects of this type of collision have not been developed. Plane-wave theory has been used in several approximate treatments [2, 3, 4]. One of the unknowns encountered in the use of plane-wave theory for solid-against-liquid collisions was the particle velocity in the compressed zones.

During collision between a solid rod A having flat ends and moving with velocity *V* in the (+z)-direction of a stationary coordinate system (fig. 1) and a similar liquid rod B that is at rest, there is a radial flow of liquid at the impacted end of rod B. In order that the rods remain in contact while the compressional waves initiated by the collision move through them, the interface velocity (*I*) must obey the inequality *V−v′ >v* where *v′, v* are the particle velocities in the compressed zones.

We can then write *α*(*V−v′*)*=v* where *α* is a dimensionless coefficient having a value less than one, and

$$v+\alpha v^{\prime }=\alpha V.$$ (1)

Using the relation that exists between stress and particle velocity for plane waves, the equality of stresses at the surfaces of contact is given by

$$zv=z^{\prime }v^{\prime },$$ (2)

where *z* is the acoustic impedance (product of sound speed and density). From eq (1) and (2), the particle velocities *v*, *v*′ are found to be

$$v=\alpha z^{\prime }V/\left(z^{\prime }+\alpha z\right)$$ (3)

$$v^{\prime }=\alpha zV/\left(z^{\prime }+\alpha z\right),$$ (4)

and the plane-wave stress *σ* is given by

$$\sigma =\sigma^{\prime }=\alpha zz^{\prime }V/\left(z^{\prime }+\alpha z\right).$$ (5)

The quantity that must be determined to make these equations useful is the coefficient *α.*

One of the approximate treatments in which plane-wave theory was used for solid-against-liquid collisions [3] provides a means of deducing an explicit expression for the coefficient *α.* In this treatment the complicated situation of collision between a moving target plate and a relatively stationary liquid drop was idealized as the simple case of the collision of two rods with flat ends. If a plate is fired against a drop (fig. 1), a core of material extending through the plate under the contact area is slowed down with respect to the remainder of the plate and a similar core of material through the drop is set in motion. The cores were regarded as true cylinders free to move in the *z*-directions (fig. 1) but restrained laterally. The compressional waves that move through the cylinders were regarded as plane waves.

With use of this simple model, an equation was developed that gives pit depth *δ*′ as a function of impingement velocity *V* for collisions of metal target plates with liquid drops [3]. For impingement velocities for which elastic recovery of the plate is complete, the pit depth was taken to be the product of a numerical constant, the particle velocity given to the cylindrical core of material under the collision area, and the time that the particle velocity exists. The particle velocity was taken to be *zV/*(*z+z′*), which is the plane-wave particle velocity for solid-against-solid collisions. The time during which the particle velocity exists was taken to be 2*d/c* where *d* is the diameter of the drop and *c* is the sound speed of the liquid of which it is composed. Therefore, *δ*′ = (constant) (*d/c*) [*zV*/*(z+z*′)].

The pit-depth equation that was developed was applied first to collisions of mercury drops and waterdrops with target plates of copper, 1100–O aluminum, 2024–O aluminum, steel, and lead. The constant was found empirically to be 7.2. The equation was then found to apply without change of the constant to collisions between metal target plates and soft ductile metal spheres that flowed during and as a result of the collision.

The same equation was later applied [4] to collision of steel spheres against target plates of 1100–O aluminum, 2024–O aluminum, and copper. It was found empirically that if the target plate was struck by a rigid hardened steel sphere that did not flow as a result of the collision the constant was 17.5.

The constants found for the pit-depth equation for the case that a target plate collides with a liquid drop or soft ductile metal sphere and for the case that it collides with a rigid hardened steel sphere provide a means of obtaining an explicit expression for the coefficient *α.* The two cases differ only in the particle velocity given to the core of material through the target plate. The particle velocity *v*′ for solid-against-solid collisions was used in each case. The particle velocity *v*′ for solid-against-liquid collisions should have been used for the case that the target plate collided with a liquid drop or with a soft deforming metal sphere that would flow as a result of the collision.

Because it is the particle velocity given to the core of target material under the collision area that is different, and because the constant 7.2 is 0.41 of the constant 17.5, it follows that *αzV*/(*z′+αz*) = 0.41 *zV*/(*z′+z*) from which

$$\alpha =0.41/[1+(0.59\;z/z^{\prime })].$$ (6)

Values of *α* calculated with use of eq (6) for collisions of waterdrops and mercury drops with target plates of aluminum, copper, lead, and glass are given in table 1. It can be seen that the value of *α* for waterdrop collisions with the solid materials cited is very close to 0.4. This is in exact agreement with an independent determination of *α* made earlier [2]. It was found experimentally [2] that 0.00118 sec were required for a glass plate to move through a 0.57-cm-diam waterdrop when the relative impingement velocity was 820 cm/sec (26.9 ft/sec). The velocity at which the plate moved through the drop was 484 cm/sec. It was assumed that no particle velocity was given to the cylinder of glass through the plate under the collision area. Then the velocity at which the plate moved through the drop was (1−*α*)*V.* To this degree of approximation (1−*α*)820 = 484 and *α* = 0.4.

In consideration of this independent evaluation of the coefficient *α* for waterdrop collisions, it appears, in retrospect, that had the proper particle velocity been used in [3], the numerical constant found empirically for the equation to calculate the depth of pits produced by collision of a metal plate with liquid drops would have been the same as that with rigid steel spheres, namely, 17.5 [4].

## Figures and Tables

**Figure 1:**
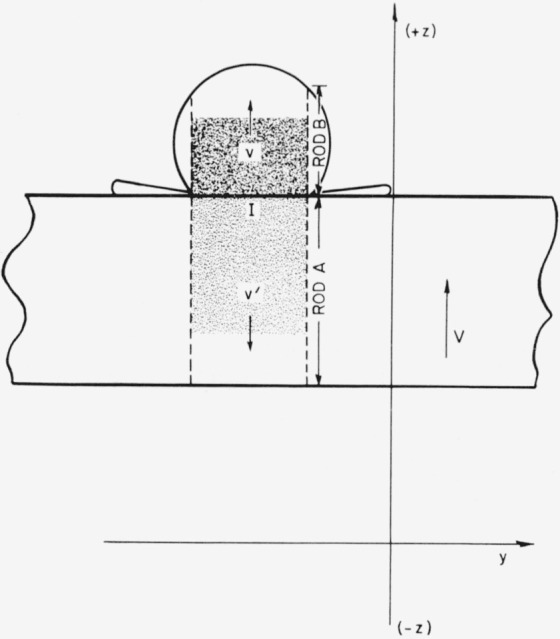
Collision between a plate moving at velocity V and a liquid drop at rest idealized as collision between a solid rod A and a liquid rod B.

**Table 1:** Some values of the coefficient α

Target	Aluminum	Copper	Lead	Glass
Drop				
				
Water	0.39	0.40	0.40	0.38
Mercury	.24	.32	.28	.22

## Footnotes

1 Figures in brackets indicate the literature references at the end of this paper.
